# Cofactor maturase NifEN: A prototype ancient nitrogenase?

**DOI:** 10.1126/sciadv.ado6169

**Published:** 2024-06-12

**Authors:** Chi Chung Lee, Kamil Górecki, Martin Stang, Markus W. Ribbe, Yilin Hu

**Affiliations:** ^1^ Department of Molecular Biology and Biochemistry, University of California, Irvine, CA 92697- 3900, USA.; ^2^ Department of Chemistry, University of California, Irvine, CA 92697-2025, USA.

## Abstract

Nitrogenase plays a key role in the global nitrogen cycle; yet, the evolutionary history of nitrogenase and, particularly, the sequence of appearance between the homologous, yet distinct NifDK (the catalytic component) and NifEN (the cofactor maturase) of the extant molybdenum nitrogenase, remains elusive. Here, we report the ability of NifEN to reduce N_2_ at its surface-exposed L-cluster ([Fe_8_S_9_C]), a structural/functional homolog of the M-cluster (or cofactor; [(*R*-homocitrate)MoFe_7_S_9_C]) of NifDK. Furthermore, we demonstrate the ability of the L-cluster–bound NifDK to mimic its NifEN counterpart and enable N_2_ reduction. These observations, coupled with phylogenetic, ecological, and mechanistic considerations, lead to the proposal of a NifEN-like, L-cluster–carrying protein as an ancient nitrogenase, the exploration of which could shed crucial light on the evolutionary origin of nitrogenase and related enzymes.

## Teaser

The nitrogenase cofactor maturase NifEN is capable of N_2_ reduction and may represent a prototype ancient nitrogenase.

## INTRODUCTION

**Fig. 1.**
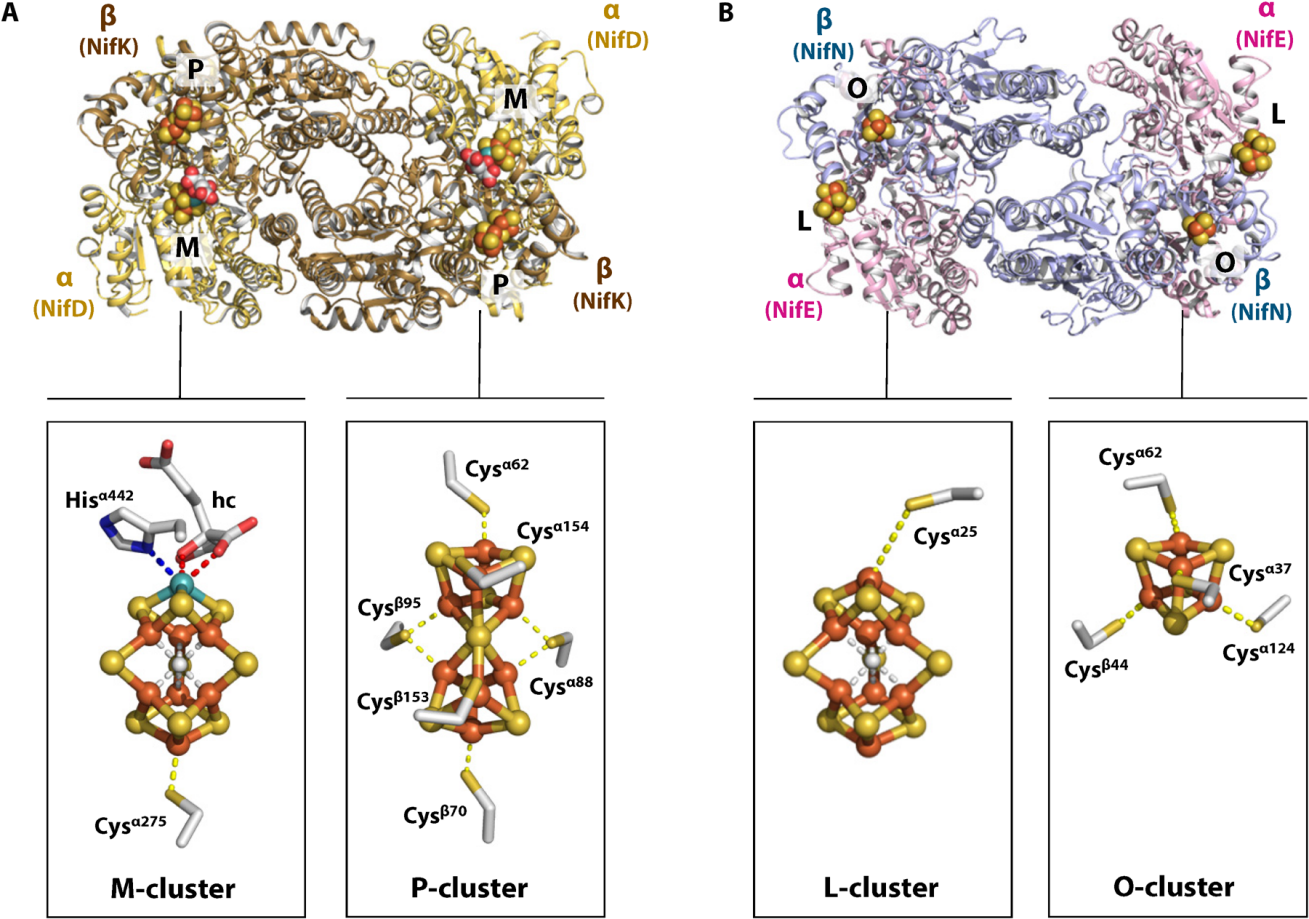
Structures of NifDK and NifEN from the Mo-dependent nitrogenase system of *Azotobacter vinelandii*. Crystal structures of the heterotetrameric *A. vinelandii* NifDK [(**A**), Protein Data Bank (PDB) entry 3U7Q] and NifEN [(**B**), PDB entry 3PDI] and their associated metalloclusters. The subunits of NifDK (A) and NifEN (B) are shown as ribbons and colored as follows: NifD (α subunit), light orange; NifK (β subunit), brown; NifE (α subunit) light purple; NifN (β subunit), blue. The M-cluster ([(*R*-homocitrate)MoFe_7_S_9_C]; PDB entry 3U7Q), P-cluster ([Fe_8_S_7_]; PDB entry 1M1N), L-cluster ([Fe_8_S_9_C]; PDB entry 3PDI), and O-cluster ([Fe_4_S_4_] cluster; PDB entry 3PDI) are shown in ball-and-stick presentation, with their ligands shown as sticks and atoms colored as follows: Fe, orange; Mo, cyan; S, yellow; C, light gray. hc, homocitrate.

Nitrogen fixation, a crucial biogeochemical process that converts N_2_ to NH_3_, is ancient (*1*, *2*). The emergence of this event has been placed in the middle of the Archean eon, around 3.8 Ga (giga annum or billion years) to 3.5 Ga ago (*3*), greatly preceding the Great Oxygenation Event (GOE) that is believed to have occurred around 2.4 Ga to 2.2 Ga ago (*4*). Yet, the evolution history of nitrogenases, a family of metalloenzymes responsible for the biological nitrogen fixation process, has remained an active topic of discussion. Three homologous nitrogenases have been identified to date: the *nif*-encoded, Mo-dependent nitrogenase; the *vnf*-encoded, V-dependent nitrogenase; and the *anf*-encoded, Fe-only nitrogenase (*5*, *6*). While it was initially proposed that the Anf system appeared first in evolution, subsequent phylogenetic studies strongly pointed to the Nif system as the ancestral species that occurred before the Vnf and Anf systems (*7*–*9*). Similar disputes arose over the evolutionary sequence of two distinct members of the Nif system: one of them, designated NifDK, is the catalytic component of the Nif system (*10*, *11*) that catalyzes the reduction of N_2_ at its active-site Mo-containing cofactor (i.e., the M-cluster; Fig. 1A and fig. S1A); the other, designated NifEN, is the cofactor maturase of the Nif system (*5*, *12*) that converts an all-Fe cofactor precursor (i.e., the L-cluster; Fig. 1B and fig. S1B) to a Mo/homocitrate-containing M-cluster before delivering the latter to the active site of NifDK. Phylogenetically established to have arisen from a single-gene duplication event, both NifDK and NifEN have been suggested as precursors to each other during evolution (*2*, *13*, *14*). Both suggestions are reasonable and supported by phylogenetic evidence; however, they revolve around an apparent paradox, that is: If NifDK was the ancestral species, then how did this enzyme assemble its complex cofactor without a maturase? Conversely, if NifEN preceded NifDK in evolution, then how did this maturase acquire the challenging nitrogen-fixing activity? Compounding the issue further was the limited bioavailability of Mo before GOE, making it particularly unlikely for a NifDK-like species with a Mo-containing cofactor to be the original species that gave rise to nitrogenase.

Recently, it was suggested that a cofactor maturase–like protein capable of reducing “easier” substrates (e.g., C_2_H_2_) (*15*), such as the L-cluster–bound NifEN (*12*), could serve as the common precursor to both NifEN and NifDK (*14*). By distinguishing the cofactor maturases from the catalytic nitrogenases on the basis of conserved ligand(s) for the NifEN-associated, all-Fe cofactor analog (i.e., the L-cluster) instead of those for the NifDK-associated, Mo-containing cofactor (i.e., the M-cluster), a strong case was made for a maturase-like, non-N_2_-reducing protein being the phylogenetic root of the extant NifEN and NifDK during evolution (*14*). This proposal is appealing, as it circumvents the problem of Mo bioavailability on early Earth and provides, at least in part, a solution to the “chicken-and-egg” paradox regarding the placement of NifEN and NifDK on an evolutionary time scale. At the same time, key questions remain as to how and why a maturase-like protein with no nitrogen-fixing activity acquired such a complex function and whether it is feasible to place this nonfunctional predecessor and the subsequent NifDK/NifEN duplication event even earlier in time than that established for the rise of nitrogen fixation at 3.8 Ga ago.

Intuitively, it is difficult to overlook the notable structural resemblance between the L-cluster and the M-cluster, particularly with respect to the conservation of their respective metal-sulfur cores that consist of two [M_4_S_3_] subcubanes (M = Fe or Mo) ligated by an interstitial carbide and three labile belt sulfides (Fig. 1) (*10*). Given the recent suggestion that the binding of nitrogenase ligand (CO) or substrate (N_2_) occurs via displacement of the belt sulfide(s) under turnover conditions (*16*–*18*), it is likely that such a unique core geometry, which is absent from any other known metalloclusters, dictates the minimum requirement for N_2_ binding and reduction. It is conceivable, therefore, that the extant, L-cluster–bound cofactor maturase (e.g., the *Azotobacter vinelandii* NifEN protein) represents an evolutionary relic of the primitive nitrogenase that gave rise to the M-cluster–bound catalytic component (e.g., the *A. vinelandii* NifDK protein) of the present-day Mo-nitrogenase. Moreover, it can be expected that such an evolutionary relic reduces N_2_ at a very low efficiency due to the imperfect composition (i.e., missing Mo and homocitrate) and unoptimized location (i.e., at the protein surface) of its cofactor species, as well as the weaker capacity of its P-cluster equivalent (i.e., the O-cluster, a [Fe_4_S_4_] cluster) in shuttling low-potential electrons to the cofactor.

## RESULTS

### Reduction of N_2_ by an O- and L-cluster–bound form of NifEN

**Fig. 2.**
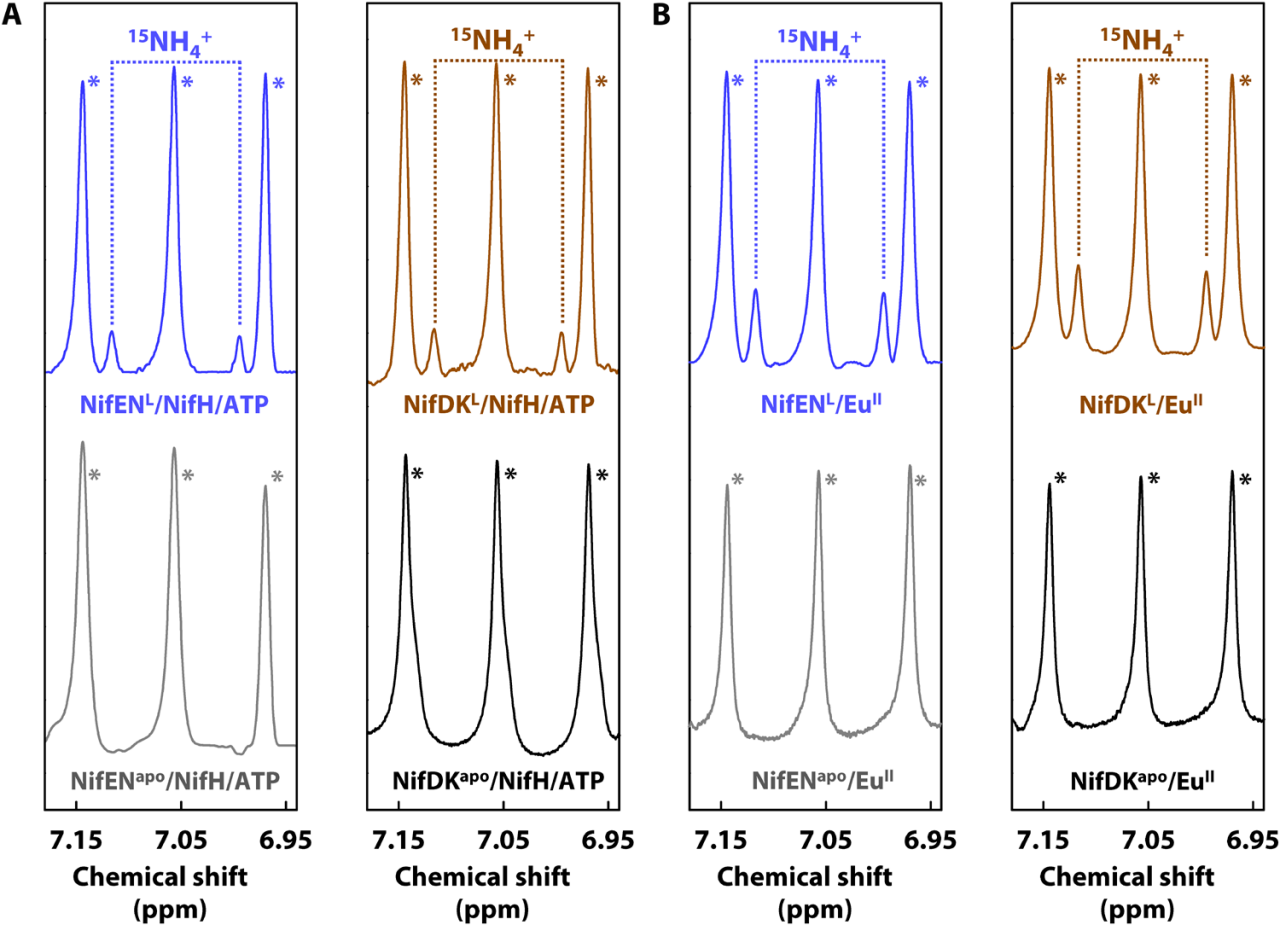
Formation of NH_4_^+^ from N_2_ reduction by NifEN- and NifDK-associated L-clusters. Frequency-selective ^1^H NMR spectra of NH_4_^+^ generated upon turnover of isotopically labeled ^15^N_2_ in (**A**) an ATP-dependent assay driven by NifH, ATP, and dithionite; and (**B**) an ATP-independent assay driven by Eu(II) DTPA. The L-cluster–bound forms of NifEN (designated NifEN^L^; blue) and NifDK (designated NifDK^L^; brown) generate ammonia from ^15^N_2_ reduction under ATP-dependent and ATP-independent conditions as indicated by the appearance of the ^15^NH_4_^+^-specific doublet at ~6.97 and ~7.12 ppm. The total turnover numbers (TON: nmol NH_4_^+^/nmol L-cluster) of these experiments are the following: (A), left: 0.8 ± 0.1; (A), right: 1.6 ± 0.3; (B), left: 1.3 ± 0.1; (B), right: 3.2 ± 0.3. In contrast, the L-cluster–deficient forms of NifEN (designated NifEN^apo^; gray) and NifDK (designated NifDK^apo^; black) do not show N_2_ rededuction as indicated by the absence of the doublet signal. Note that the triplet signals in the NMR spectra (labeled with *) represent the ^14^NH_4_^+^ background generated upon protein degradation. Control experiments conducted in the absence of ATP or in the presence of natural abundance ^14^N_2_ are shown in fig. S2, and those conducted with the solvent-extracted L-cluster alone in the presence or absence of ATP are shown in fig. S3.

Frequency-selective pulse nuclear magnetic resonance (NMR) analysis revealed the formation of ^15^NH_4_^+^, as reflected by the doublet at ~6.97 and ~ 7.12 parts per million (ppm), upon reduction of ^15^N_2_ by the L-cluster–containing NifEN from *A. vinelandii* (designated NifEN^L^) when this protein was paired with NifH, adenosine 5′-triphosphate (ATP), and dithionite (Fig. 2A, left, blue) in a reaction analogous to that catalyzed by the two-component Mo-nitrogenase. No ^15^NH_4_^+^ was detected when the L-cluster–deficient apo NifEN from *A. vinelandii* (designated NifEN^apo^) was used instead of NifEN^L^ (Fig. 2A, left, gray) or when ATP was omitted from the reaction (fig. S2A, left, top), confirming the NifEN-associated L-cluster as the origin of the N_2_-reducing activity while verifying NifH as the ATP-dependent electron donor for this reaction. Notably, ^15^NH_4_^+^ was also detected as a product of ^15^N_2_ reduction by NifEN^L^ when the reaction was driven by a strong chemical reductant, Eu(II) diethylenetriamine pentaacetic acid [Eu(II)DTPA] (Fig. 2B, left, blue). In comparison, the formation of ^15^NH_4_^+^ was not detected when the L-cluster–deficient NifEN^apo^ was combined with Eu(II) DTPA (Fig. 2B, left, gray), again correlating the NifEN-associated L-cluster to the N_2_-reducing activity in this reaction. The ability of NifEN^L^ to perform both ATP-independent and ATP-dependent N_2_ reduction points to a plausible evolutionary scenario wherein the predecessor of nitrogenase was a stand-alone, NifEN^L^-like enzyme that used electron donors (e.g., ferredoxins) to directly supply electrons to the surface-exposed cofactor and that this enzyme eventually evolved into a two-component system that used specific adenosine triphosphatase-dependent NifH as an obligate electron donor for the catalytic NifDK component to enable a high-efficiency mechanism of N_2_ reduction.

Curiously, a closer examination of the primary sequences has revealed that many of the extant NifEN sequences have both L- and P-cluster ligands (fig. S4, group II). Specifically, these NifEN species contain the same, conserved Cys ligand (Cys^α25^) of *A. vinelandii* NifEN for the surface coordination of an L-cluster–like cofactor species (fig. S4A) (*12*), and they clearly lack the key residue(s) defining a typical cofactor site of *A. vinelandii* NifDK, including those for the insertion (*19*), ligation, and reactivity (*20*) of the M-cluster (fig. S4A). However, contrary to *A. vinelandii* NifEN that contains four Cys ligands for the coordination of a subunit-bridging O-cluster (i.e., a single [Fe_4_S_4_] cluster), these NifEN species contain six Cys ligands that could accommodate either an 8Fe P-cluster (i.e., [Fe_8_S_7_]) or its 8Fe analog (e.g., a [Fe_4_S_4_] cluster pair) at the subunit interface (fig. S4B). Such a NifEN species is yet to be isolated and characterized, although it is anticipated to have a hybrid cluster composition with a surface-exposed L-cluster (found in the extant NifEN) and a subunit-bridging P-cluster or its analog (found in the extant NifDK) and, therefore, have a certain degree of N_2_-reducing activity.

### Generation of a P- and L-cluster–bound form of NifDK

**Fig. 3.**
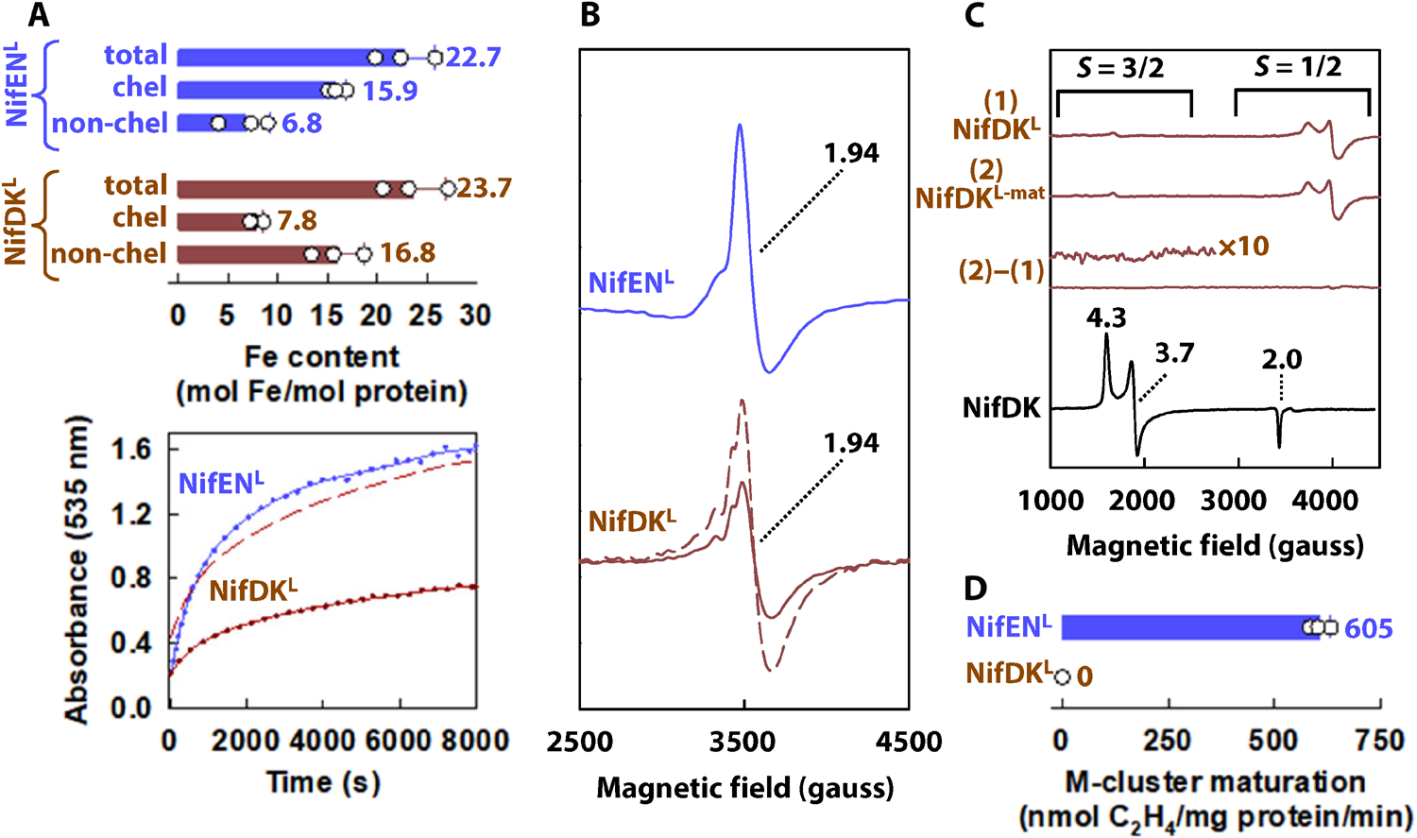
Biochemical and spectroscopic features of NifEN^L^ and NifDK^L^. (**A**) Top: Total Fe (“total”) versus chelated Fe (“chel”; originating from the surface-exposed L-cluster) and non-chelated Fe (“non-chel”; derived from the difference between “total” and “chel”) upon treatment of NifEN^L^ (blue) and NifDK^L^ (brown) with bathophenanthroline disulfonate (BPS), an Fe chelator. The amount of chelated Fe (*n* = 3) was calculated on the basis of a molar extinction coefficient of 22,140 M^−1^ cm^−1^. Bottom: Kinetics of the L-cluster–specific Fe chelation upon incubation of BPS with NifEN^L^ (blue line-and-dots) and NifDK^L^ (brown line-and-dots), monitored by the time-dependent formation of the BPS-Fe complex at 535 nm. Upon normalization of the L-cluster content, NifDK^L^ (brown dashed line) shows a highly similar kinetic behavior of Fe chelation to its NifEN^L^ counterpart (blue line-and-dots). (**B**) Electron paramagnetic resonance (EPR) spectra of indigo disulfonate (IDS)–oxidized NifEN^L^ (solid blue) and NifDK^L^ (solid brown). Upon normalization of the L-cluster content, the spectra of NifDK^L^ (dashed brown) and NifEN^L^ (solid blue) are highly similar in line shape and magnitude, particularly with respect to the L-cluster–specific *g* = 1.94 feature. Quantitation of the spectra (B) with a Cu(II) standard under nonsaturating conditions revealed spin concentrations of 53 and 28 μM, respectively, for NifEN^L^ (10 mg/ml) and NifDK^L^ (10 mg/ml), which align well with the L-cluster–specific Fe chelation data (A). The NifEN^apo^ and NifDK^apo^ controls are EPR-silent upon IDS oxidation (see fig. S5). (**C**) EPR spectra of dithionite-reduced (i) NifDK^L^ and (ii) NifDK^L-mat^. Prepared by re-isolating NifDK^L^ after incubation with the M-cluster maturation factors (i.e., NifH, ATP, MoO_4_^2−^, homocitrate, and dithionite), NifDK^L-mat^ does not display the M-cluster–specific signal at *g* = 4.3, 3.7, 2.0. (**D**) M-cluster maturation activities of NifEN^L^ (blue) and NifDK^L^ (brown). Contrary to NifEN^L^ that can serve as an M-cluster donor upon maturation of its associated L-cluster, NifDK^L^ cannot transform its associated L-cluster to an M-cluster for the subsequent reconstitution of NifDK^apo^ (*n* = 3).

To test our hypothesis, we incubated a P-cluster–containing, yet M-cluster–deficient apo NifDK from *A. vinelandii* (designated NifDK^apo^) with the solvent-extracted L-clusters (from NifEN^L^) and subsequently repurified the L-cluster–bound NifDK protein (designated NifDK^L^) for analysis. While metal analysis revealed a 50% L-cluster occupancy of NifDK^L^ relative to that of its NifEN^L^ counterpart (Fig. 3A, top), NifDK^L^ displayed an L-cluster–specific, *g* = 1.94 EPR signal highly similar to that displayed by NifEN^L^ in the indigo disulfonate (IDS)–oxidized state (Fig. 3B) (*21*), as well as comparable kinetics of Fe chelation when treated with an Fe chelator, bathophenanthroline, upon normalization of the L-cluster content (Fig. 3A, bottom). Supported further by the presence of analogous, conserved Cys ligands on the surface of both NifEN (Cys^α25^) and NifDK (Cys^α45^) (fig. S4A) (*12*), these observations suggest that the L-cluster is attached at a surface-exposed location in NifDK^L^ analogous to that of the L-cluster in NifEN^L^, rendering NifDK^L^ resemblant to NifEN^L^ with respect to the cofactor site but distinct with respect to the P-cluster site (fig. S6).

### Reduction of N_2_ by a P- and L-cluster–bound form of NifDK

Excitingly, NifDK^L^ demonstrated the ability to perform both ATP-dependent (Fig. 2A, right, brown) and ATP-independent (Fig. 2B, right, brown) reduction of ^15^N_2_ to ^15^NH_4_^+^, and control experiments firmly established the NifDK-associated L-cluster as the origin of this activity (Fig. 2, A and B, right, black). Upon normalization of the L-cluster content, NifDK^L^ showed a higher N_2_-reducing activity than NifEN^L^, consistent with a higher efficiency of its P-cluster species to shuttle electrons for catalysis (see legend of Fig. 2 for turnover numbers). Yet, contrary to NifEN^L^, NifDK^L^ was unable to support maturation of the L-cluster to an M-cluster upon incubation with the full complement of cofactor maturation factors (i.e., NifH, ATP, MoO_4_^2−^, homocitrate, and dithionite), as evidenced by the inability of thus-treated protein to display the M-cluster–specific EPR signal (Fig. 3C) or carry out substrate reduction (Fig. 3D).

### An L-cluster–bound, NifEN-like protein: A predecessor of the present-day nitrogenase?

Given that the L-cluster is a common denominator of NifEN^L^ and NifDK^L^, the observation that both proteins show N_2_-reducing activities points to the possible existence of an ancient nitrogenase during evolution that carried an L-cluster–like, all-Fe cofactor at the surface of a NifEN-like protein scaffold. It is possible that such a predecessor gave rise to both NifDK and NifEN of the present-day Mo-nitrogenase via concomitant evolvement of its two cluster sites into either a catalytic P/M configuration (in NifDK) or a biosynthetic O/L configuration (in NifEN). While arguments can be made on the basis of phylogenetic analyses (fig. S4) (*14*) for the presence of either a P-cluster (coordinated by three Cys ligands in the α subunit and three Cys ligands in the β subunit) or an O-cluster (coordinated by three Cys ligands in the α subunit and one Cys ligand in the β subunit) in this NifEN-like predecessor, the first scenario is more plausible, as it could be explained by an initial gene duplication that resulted in the highly homologous α and β subunits (each carrying three Cys ligands), followed by mutation (or loss) of two Cys ligands in the β subunit. Such a scenario would be simpler and, therefore, more feasible than that with the two non-Cys residues in the β subunit reverting back to two Cys residues. It implies that the loss of the P-cluster may be crucial for evolving the predecessor toward the specialized function in cofactor assembly (i.e., the L- to M-cluster maturation by the extant NifEN); conversely, maintaining the P-cluster would be necessary for evolving the predecessor toward a higher efficiency in substrate reduction (i.e., the reduction of N_2_ by the extant NifDK).

**Fig. 4.**
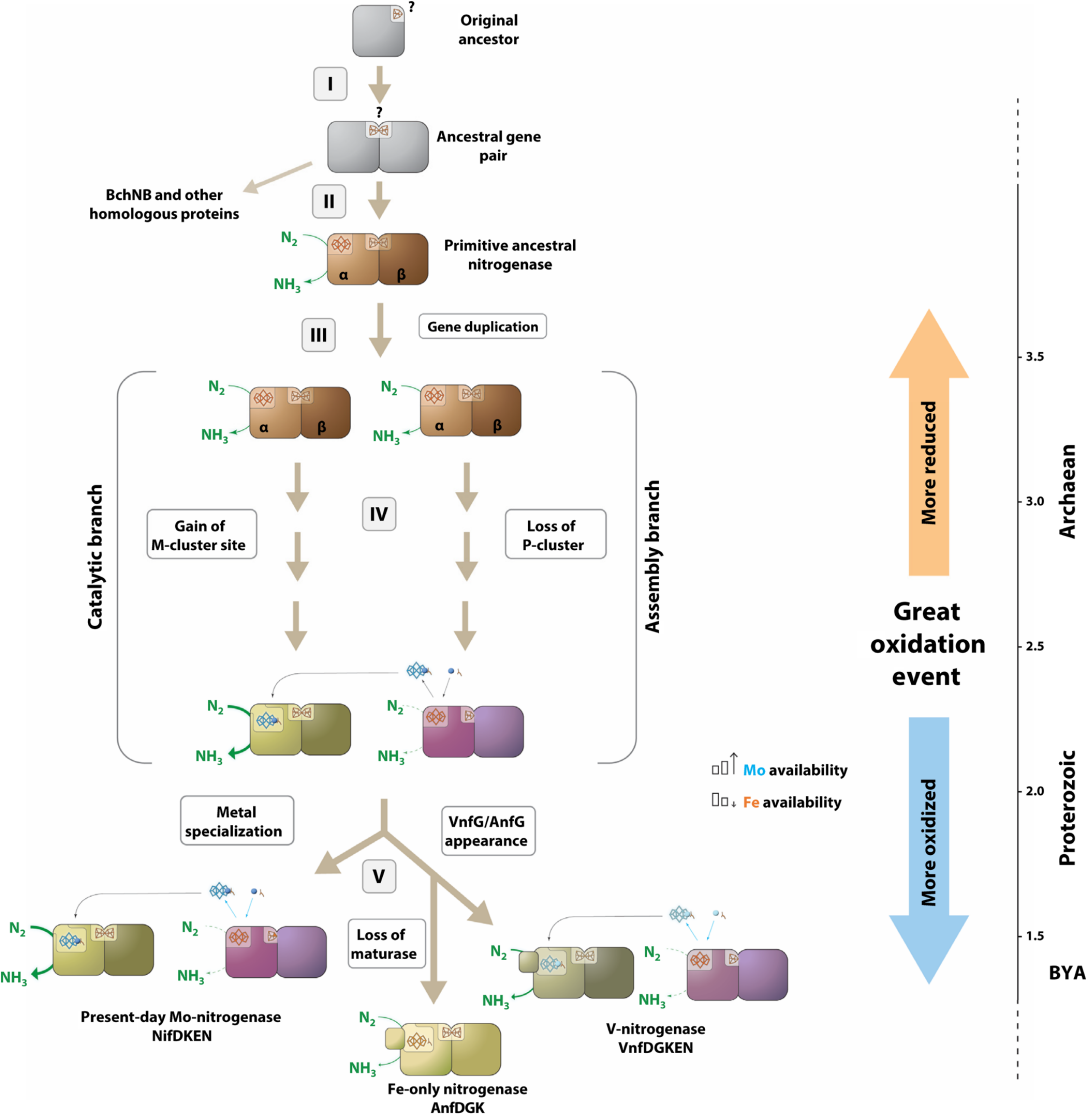
Proposed evolutionary history of nitrogenase. An ancestral gene duplication event gave rise to the α and β subunits and, given their closer similarities, this event likely occurred before their divergence into nitrogenase and related proteins (e.g., BchNB) (I). The primitive nitrogenase emerged from this ancestral gene pair, featuring an L-cluster for nitrogen fixation and a P-cluster for electron transport (II). A second gene duplication of the αβ gene pair permitted the exploration of new evolutionary space while allowing improvement of the nitrogen-fixing activity (III). One gene copy evolved into a specific maturase for the incorporation of Mo/homocitrate into the L-cluster (assembly branch) and the other copy evolved into an efficient catalyst that enclosed the M-cluster at a unique reaction site for nitrogen fixation (catalytic branch) (IV). Specialization of nitrogenase and maturase continued post-GOE due to a shift in metal availability, resulting in the appearance of NifDK (the catalytic component of the extant Nif system) and NifEN (the cofactor maturase of the extant Nif system). Subsequently, the Vnf and Anf systems evolved for adaptation to specific metals, with Vnf likely appearing first, followed by Anf from Vnf gene duplication but without duplication of the maturase (V). This proposal places the emergence of nitrogen fixation at least three billion years ago, coinciding with the shift of metal availability during the GOE while underscoring the evolutionary advantage and adaptability of early nitrogenases in metal-scarce environments.

A proposal (Fig. 4) can be made on the basis of our phylogenetic and experimental data (see Figs. 2 and 3 and fig. S4), which involves a first gene duplication event (Fig. 4, I) that gave rise to the α and β subunits of an ancestral “Fe-only nitrogenase” that was clearly distinct from the extant Anf system (Fig. 4, II). This step was followed by a second gene duplication event (Fig. 4, III) and the subsequent evolution of one copy into NifDK (catalytic component) and the other copy into NifEN (cofactor maturase) of the modern NifDK system (or the Mo-nitrogenase) (Fig. 4, IV and V). The ancestral Fe-only nitrogenase had a NifEN-like protein scaffold with a P-cluster or an 8Fe analog of this cluster bridged between its α and β subunits and an L-cluster–like, Fe-only cofactor attached at the surface of the α subunit. Despite the low N_2_-reducing activity of this primitive nitrogenase, under early Earth conditions where fixed nitrogen was the limiting nutrient for the biosphere, even a very poorly functioning nitrogenase would provide a major evolutionary advantage to its host organism over other organisms. The second gene duplication event, on the other hand, would not have given the organism having two gene copies of the primitive nitrogenase a selective edge in the beginning. Yet, as has been demonstrated for countless gene duplication events, having two copies of the same gene(s) allows an organism to retain the original functionality with one copy of gene product(s) while allowing it to probe the evolutionary space with the other, thus incurring no penalties (*22*, *23*). It is possible, therefore, that in the case of nitrogenase, the first copy of Nif gene products remained as an N_2_-reducing enzyme (Fig. 4, “catalytic branch”) by maintaining its P-cluster while evolving its cofactor site for improved efficiencies of electron transfer and substrate reduction, whereas the second copy of Nif gene products evolved toward a specialized maturase (Fig. 4, “assembly branch”) by losing half of its P-cluster while developing a mechanism for incorporating Mo (alongside homocitrate) into the primitive Fe-only cofactor and subsequently delivering it to the well-defined cofactor site within the nitrogenase enzyme that was derived from the parallel evolution of the first copy of Nif genes.

Given the absence of the atmospheric O_2_ and the presence of Fe-rich oceans before the emergence of oxygenic photosynthesis (*4*), it is plausible that in the early stages of evolution, the regulation of nitrogenase expression in a nitrogen-fixing organism might rely solely on its need for fixed nitrogen (i.e., NH_3_) and that the metal usage by nitrogenase in this organism could be either Mo or Fe, depending on the availability of Mo. Dating back to the last universal common ancestor, the existence of molybdoproteins suggests certain bioavailability of Mo—albeit low—in early Earth’s history. However, utilization of Mo by nitrogenase is quite distinct from that by other molybdoproteins, suggesting that this event might have evolved separately from the “standard” Mo metabolism where molybdoproteins are concerned. Isotopic fractionation measurements have placed the emergence of Mo-based nitrogen fixation at 3.2 Ga, at the latest (Fig. 4, IV) (*24*). Such a timeline would give the first diazotrophs plenty of time to evolve high affinity/efficacy Mo uptake systems (or scavengers like molybdophores) and perfect the Mo insertion mechanism. Once evolved, the “conventional” Mo-containing nitrogenase would take over because of its high efficiency, thereby offering the host organisms an enormous evolutionary advantage over their competitors when the availability of fixed nitrogen was a rate-limiting factor for cell growth. Later, the “alternative” V- and Fe-only nitrogenases would evolve via additional gene duplication events (Fig. 4, V), thereby accomplishing a tighter regulation of nitrogenase expression through metal availability (Mo > V > Fe) along with other key environmental conditions, such as oxygen tension and the availability of the fixed nitrogen, which control the expression of the present-day nitrogenase systems.

## DISCUSSION

Apart from phylogenetic and ecological considerations, it is also tempting to envision the evolution history of biological nitrogen fixation in the mechanistic context of the modern-day nitrogenase enzyme. Substrate reduction by the Mo-nitrogenase has come a long way from that of our proposed, primitive Fe-only nitrogenase from the perspectives of both the cofactor species and the protein scaffold, highlighting the intricacy of metalloprotein evolution where these two elements intertwine with each other. With respect to the cofactor, while its core geometry remained intact as exemplified by the three catalytically indispensable belt sulfides, a heterometal (Mo) was inserted along with an organic moiety (homocitrate) at one “end” of the primitive all-Fe cofactor, thereby modifying its redox properties while introducing an asymmetric element that is crucial for its structure and function (fig. S7). Accompanying such a change in the cofactor, the protein scaffold evolved toward that with a well-protected cofactor binding pocket “buried” within the peptides, with a pair of ligands (a His ligand and a Cys ligand) implemented for the position-specific coordination of the two “ends” of the cofactor (Mo and Fe), now distinguishable due to the asymmetry introduced via Mo/homocitrate incorporation (fig. S7). Breaking the threefold symmetry of the cofactor further, the local protein environments evolved toward those with disparate proton-donating abilities for the three belt-sulfur locations, which could, in principle, allow for a sequential protonation/reduction of N_2_ at the three locations.

Such a proposal has been put forward recently based on our combined structural and biochemical studies of the Mo-nitrogenase under turnover conditions (*17*, *18*). This mechanistic model (fig. S8A) involves binding of N_2_ via displacement of one belt sulfur, followed by rotation of the cofactor for the stepwise reduction N_2_ to NH_3_ at the other two belt-sulfur locations and, subsequently, the release of NH_3_ via belt-sulfur replacement. In addition, it posits an alternating docking of NifH on the two αβ dimers of NifDK that drives an asynchronous, ATP-dependent cofactor rotation in these two dimers, thereby permitting the same sequence of events to occur in the two cofactors but one step apart from each other (fig. S8B). Consistent with the cryo–electron microscopy structure of a turnover Mo-nitrogenase complex with a 1:1 molar ratio of NifH and NifDK (*25*), the proposal of an alternating docking of NifH on NifDK highlights yet another asymmetric element introduced during the course of nitrogenase evolution, with the plausible evolution of a single-component, primitive nitrogenase to a two-component, NifH/ATP-dependent system accompanied by further differentiation of the two cofactors of NifDK via an alternating interaction of NifH with the respective dimers of NifDK housing these cofactors (fig. S8B). The asymmetry of the reaction sequences introduced upon alternating docking of NifH/ATP, along with the asymmetry of the two reaction sites in NifDK that is defined by the locations of the three cofactor belt-sulfurs, creates an energy, spatial, and temporal division for the enzyme to effectively break down the large amount of energy supplies (16 ATPs per reduced N_2_) and reducing equivalents (8 e^−^/H^+^ per reduced N_2_) required for N_2_ reduction into smaller, manageable units, thereby allowing the multiple events of this complex reaction to proceed in a stepwise, controlled manner instead of having them occur all at once, in one place.

Last but not least, the proposal of an L-cluster like, all-Fe cofactor as the primitive nitrogenase cofactor begs the question of how such a complex metallocluster came into existence during evolution. On the basis of studies of the cofactor assembly in the present-day Mo-nitrogenase (*5*, *19*), it can be assumed that the last precursor to an L-cluster–like structure was a pair of [Fe_4_S_4_] clusters. Spontaneous formation of [Fe_4_S_4_] clusters has been demonstrated and, given their ability to reduce carbon substrates like CO and CO_2_, the evolutionary precursor of these small iron-sulfur clusters could be traced to iron sulfide mineral deposits near deep-sea hypothermal vents that gave rise to “pioneer organisms” capable of autotrophic carbon fixation in the context of the iron-sulfur world model proposed by Wächtershäuser (*26*, *27*). Yet, the formation of an L-cluster–like structure from [Fe_4_S_4_] building blocks is not a spontaneous process; on the contrary, it involves radical *S*-adenosyl-L-methionine (SAM) chemistry-based coupling and rearrangement of two [Fe_4_S_4_] clusters concomitant with insertion of a SAM-derived carbide and a sulfite-derived sulfide, resulting in a structure with two [Fe_4_S_3_] subcubanes linked by three belt sulfides and an interstitial carbide (*28*, *29*). Dubbed “the heart of steel,” the six-coordinated interstitial carbide holds the cofactor structure together, thereby allowing for the dynamic belt-sulfur mobilization that is proposed to be mandatory for substrate binding and product release by nitrogenase (*17*, *18*). Hence, the emergence of biological nitrogen fixation could be intimately associated with the rise of radical chemistry, a plausible scenario given the observation that sulfate radicals could enable the spontaneous formation of a non-enzymatic Krebs cycle precursor (*30*). In this scenario, it is possible that the NifEN-like primitive nitrogenase originated from another precursor enzyme with the capability of synthesizing a complex, L-cluster–like cofactor. The questions of whether such a precursor enzyme existed before our proposed primitive nitrogenase or if it had a dual function in catalysis and assembly remain unclear, the exploration of which could shed crucial light on the evolution history of nitrogenase and related enzymes.

## MATERIALS AND METHODS

Unless otherwise specified, all chemicals were purchased from Sigma-Aldrich (St. Louis, MO) and Thermo Fisher Scientific (Waltham, MA), and all experiments were performed under an Ar atmosphere using Schlenk techniques and a glove box operating at <3 ppm O_2_.

### Cell growth and protein purification

The following *A. vinelandii* strains (*31*) were used in this study: (i) YM9A, which expresses a His-tagged, O-cluster replete, yet L-cluster–depleted form of NifEN (designated NifEN^apo^) in a *nifB-*deletion background; (ii) DJ1041, which expresses a His-tagged, O- and L-cluster replete form of NifEN (designated NifEN^L^); (iii) DJ1143, which expresses a His-tagged, P-cluster replete, yet M-cluster–depleted form of NifDK (designated NifDK^apo^) in a *nifB-*deletion background; and (iv) DJ1141, which expresses a His-tagged, P- and M-cluster replete form of NifDK (designated NifDK^M^). Each strain was grown at 30°C in 180-liter batches in a 200-liter fermenter (New Brunswick Scientific) in Burke’s minimal medium supplemented with 2 mM ammonium acetate. Cell growth was monitored by measuring cell densities at 436 nm using a Spectronic 20 Genesys spectrometer (Spectronic Instruments). Once ammonia was depleted in the medium, cells were allowed to derepress for 3 hours before being harvested by a flow-through centrifugal harvester (Cepa). Published methods were used to purify the His-tagged NifEN^apo^, NifEN^L^, NifDK^apo^, and NifDK^M^ proteins and the nontagged NifH protein (*32*, *33*).

### Generation of an L-cluster–bound NifDK

The L-clusters were extracted from NifEN^L^ into *N*-methylformamide (NMF) using a previously described protocol (*34*). Subsequently, an L-cluster–bound form of NifDK (designated NifDK^L^) was generated by mixing, in a total volume of 4.5 ml, 70 μl of the NMF-extracted L-clusters (2.8 mM), 6 mg of NifDK^apo^, 20 mM Na_2_S_2_O_4_, and 25 mM Tris-HCl (pH 8.0). The mixture was incubated at 30°C for 15 min and passed through a G25 desalting column, followed by a concentration of the NifDK^L^-containing eluant of the G25 column using an Amicon concentrator [molecular weight cutoff (MWCO): 50 kDa].

### Maturation of the L-cluster on NifEN or NifDK

Maturation of NifEN- or NifDK-bound L-cluster was carried out by mixing, in a total volume of 0.9 ml, 0.9 mg of NifEN^L^ or NifDK^L^, 0.8 mg of non-tagged NifH, 0.8 mM Na_2_ATP, 1.6 mM MgCl_2_, 10 mM creatine phosphate, 52 U of creatine phosphokinase, 0.3 mM homocitrate, 0.3 mM Na_2_MoO_4_, 20 mM Na_2_S_2_O_4_, and 25 mM Tris-HCl (pH 8.0). Each mixture was incubated at 30°C for 45 min to permit maturation of the L-cluster into an M-cluster on NifEN or, if possible, on NifDK. Subsequently, the maturation mixture containing either NifEN or NifDK was combined with 0.5 mg of NifDK^apo^, 1.5 mg of NifH, 30 mM Na_2_ATP, 75 mM MgCl_2_, 90 mM creatine phosphate, 80 U of creatine phosphokinase, 20 mM Na_2_S_2_O_4_, and 25 mM Tris-HCl (pH 8.0) into a total volume of 1 ml in a 10-ml vial that contained 10% C_2_H_2_ gas in the headspace. The reaction mixture was then incubated at 30°C for 30 min before the determination of enzymatic activities as described previously (*35*). For electron paramagnetic resonance (EPR) analysis, the maturation mixture containing NifDK was subjected to immobilized metal affinity chromatography on a nickel–nitrilotriacetic acid column (*32*, *34*) for the repurification of NifDK following the attempted maturation of its associated L-cluster (designated NifDK^L-mat^).

### Determination of iron content

The Fe contents of the NifEN^L^ and NifDK^L^ were determined by inductively coupled plasma optical emission spectroscopy using a Thermo Scientific iCAP7000. A stock solution of the elemental Fe (1 mg/ml; Thermo Fisher Scientific) was diluted to make standard solutions for the purpose of calibration. Each protein sample was mixed with 100 μl of concentrated sulfuric acid (H_2_SO_4_) and 100 μl of concentrated nitric acid (HNO_3_), followed by heating at 250°C for 30 min. This procedure was repeated until the solution became colorless. Subsequently, the solution was cooled to room temperature and diluted to a total volume of 7.5 ml with 2% HNO_3_ before Fe analysis.

### EPR spectroscopy

EPR samples were prepared in a Vacuum Atmospheres glove box filled with Ar (<3 ppm O_2_) and flash-frozen in liquid nitrogen before analysis. Reduced samples contained 10% (v/v) glycerol, 250 mM imidazole, 2 mM Na_2_S_2_O_4_, and 25 mM Tris-HCl (pH 8.0). Oxidized samples were prepared by incubating the reduced samples with 5 mM IDS for 5 min. EPR spectra were recorded by an ESP 300E spectrophotometer (Bruker) interfaced with an ESR-9002 liquid-helium continuous-flow cryostat (Oxford Instruments) using a microwave power of 50 mW, a gain of 5 × 10^4^, a modulation frequency of 100 kHz, and a modulation amplitude of 5 G. Five scans of perpendicular-mode EPR spectra were recorded for each sample at 10 K (reduced samples) or 15 K (oxidized samples) using a microwave frequency of 9.62 GHz.

### Iron chelation assays

Fe chelation assays were carried out to determine the cluster accessibility of NifEN^L^ and NifDK^L^. A mixture containing NifEN^L^ or NifDK^L^ (1 mg/ml), 2 mM Na_2_S_2_O_4_, and 25 mM Tris-HCl (pH 8) was prepared anaerobically and used to blank the UV-Vis spectrophotometer at 535 nm every 30 s. Data collection was initiated immediately upon the addition of 5 mM bathophenanthroline disulfonate, an Fe chelator. The amount of chelated Fe was calculated on the basis of a molar extinction coefficient (ε) of 22,140 cm^−1^ M^−1^ at 535 nm (*36*).

### Frequency-selective pulse NMR analysis

The formation of NH_4_^+^ was analyzed by frequency-selective pulse NMR using a protocol adapted from a previously published method (*17*). The ATP-dependent turnover reactions contained, in a total volume of 1.5 ml, 5 mg of NifEN^L^ or NifDK^L^, 12.5 mg of NifH, 100 mM Na_2_ATP, 250 mM MgCl_2_, 300 mM creatine phosphate, 250 U of creatine phosphokinase, 0.4 mM Na_2_S_2_O_4_, and 25 mM Tris-HCl (pH 8.0). Control assays had the same composition as the reactions but either contained 5 mg of NifEN^apo^ or NifDK^apo^ or did not contain Na_2_ATP, MgCl_2_, creatine phosphate, and creatine phosphokinase. The ATP-independent turnover reactions contained, in a total volume of 1.5 ml, 5 mg of NifEN^L^ or NifDK^L^, 2 mM Na_2_SO_3_, 20 mM Eu(II)-DTPA, and 25 mM Tris-HCl (pH 8.0). Control assays had the same composition as the reactions but contained 5 mg of NifEN^apo^ or NifDK^apo^. The reaction or control assay was incubated at 30°C for 30 min under a gas atmosphere of 100% ^15^N_2_ or ^14^N_2_, followed by transfer of the mixture to a 1.5-ml tube containing a Microcon Centrifugal Filter (MWCO: 10 kDa; Millipore) and removal of proteins by centrifugation at 10,000*g* for 20 min. Subsequently, 0.5 ml of each Microcon filtrate was combined with 0.05 ml of 1 M H_2_SO_4_ and 0.05 ml of CD_3_CN as a locking agent. The ^1^H NMR spectra were recorded using a Bruker AVANCE600 spectrometer equipped with a CBBFO cryoprobe. Water suppression was used, and spectra were referenced by setting the residual CH_3_CN signal to 2.06 ppm (*37*). A total of 4096 scans were recorded per sample, with an acquisition time of 1.5 s and a relaxation delay of 5 s. All spectra were background adjusted and de-sloped.

### Phylogenetic analysis

Figure S4 was generated in iTOL v. 6.7.3 (*38*) using the tree file tree1.ancnode.tre for generating the trees and the multiple sequence alignment file tree1.align_withancestors.fasta for annotations. The files were retrieved from the repository corresponding to a previous report (*14*). The tree was pruned to show only α or β subunits in the respective panels, and the outgroups (BchB and BchN) were collapsed for brevity.
