# Supplementary material for: Cofactor maturase NifEN: A prototype ancient nitrogenase?
Source: Sci Adv. 2024 Jun 12;10(24):eado6169. doi: 10.1126/sciadv.ado6169 (PMC11168457; doi:10.1126/sciadv.ado6169)
Supplement: Supplementary file 1 — Figs. S1 to S8 [file sciadv.ado6169_sm.pdf]

Science Advances
AAAS

Supplementary Materials for

Cofactor maturase NifEN: A prototype ancient nitrogenase?

Chi Chung Lee *et al.*

Corresponding author: Markus W. Ribbe, mribbe@uci.edu; Yilin Hu, yilinh@uci.edu

Sci. Adv. **10**, eado6169 (2024)
DOI: 10.1126/sciadv.ado6169

This PDF file includes:

Figs. S1 to S8

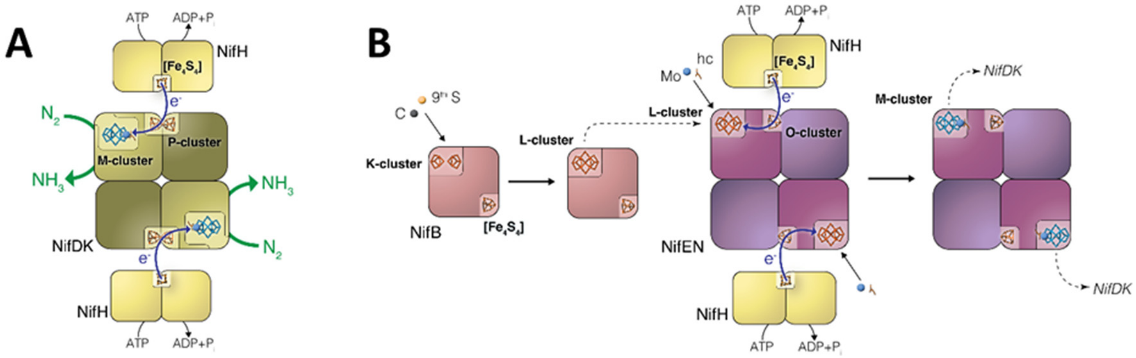

Fig. S1. Substrate reduction and cofactor maturation of Mo-nitrogenase. (**A**) The homodimeric NifH (reductase component) acts as the obligate electron donor for the heterotretrameric NifDK (catalytic component) and delivers electrons in an ATP-dependent process from its [Fe_4S_4] cluster, via the P-cluster ([Fe_8S_7]), to the M-cluster ([(*R*-homocitrate)$MoFe_7S_9C$]) of NifDK, where reduction of N_2 to NH_3 occurs. (**B**) On NifB, a pair of [Fe_4S_4] clusters (designated the K-cluster) undergo radical SAM-dependent transformation into an [Fe_8S_9C] precursor (designated the L-cluster) concomitant with the incorporation of an interstitial C and a 9th S. The L-cluster is structurally indistinguishable from the fully assembled M-cluster except for the absence of Mo and homocitrate. Once generated on NifB, the L-cluster is transferred to NifEN, where it is matured into an M-cluster upon the NifH-mediated insertion of Mo and homocitrate prior to transfer of the matured M-cluster to its target binding site in NifDK.

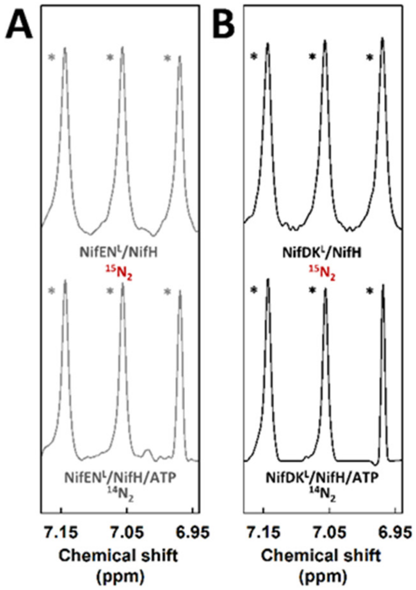

Fig. S2. Controls assays conducted without ATP or under $^{14}N_2$. Frequency-selective ^1H NMR spectra of NH_4^+ generated by control assays containing NifEN^L (**A**) or NifDK^L (**B**) along with NifH and dithionite under isotopically labeled $^{15}N_2$ (upper) or with NifH, ATP and dithionite under natural abundance $^{14}N_2$ (lower). Note that the triplet signals in the NMR spectra (labeled with *) represent the $^{14}NH_4^+$ background resulting from protein degradation.

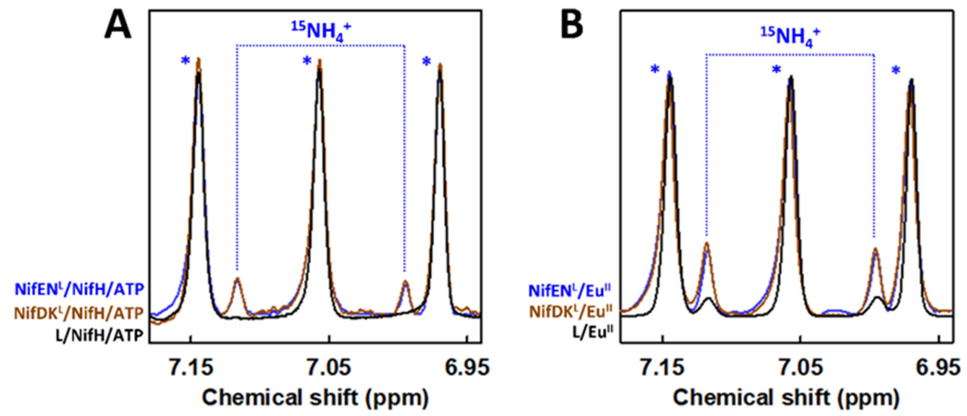

Fig. S3. Control assays conducted with the solvent-extracted L-cluster. Frequency-selective ^{1}H NMR spectra of NH_4^+ generated by control assays containing the solvent-extracted L-cluster alone (black) under ATP-dependent (**A**) or ATP-independent (**B**) conditions. Compared to $NifEN^L$ (blue) and $NifDK^L$ (brown) that reduce $^{15}N_2$ under ATP-dependent (A) and ATP-independent (B) conditions, the isolated L-cluster (black) shows no activity (A) or a minor activity (B) in N_2 reduction, as indicated by (A) the absence or (B) the minor presence of the $^{15}NH_4^+$-specific doublet at ~6.97 and ~7.12 ppm. Note that the triplet signals in the NMR spectra (labeled with *) represent the $^{14}NH_4^+$ background generated upon protein degradation. The data of assays containing $NifEN^L$ (blue) and $NifDK^L$ (brown) are taken from Fig. 2.

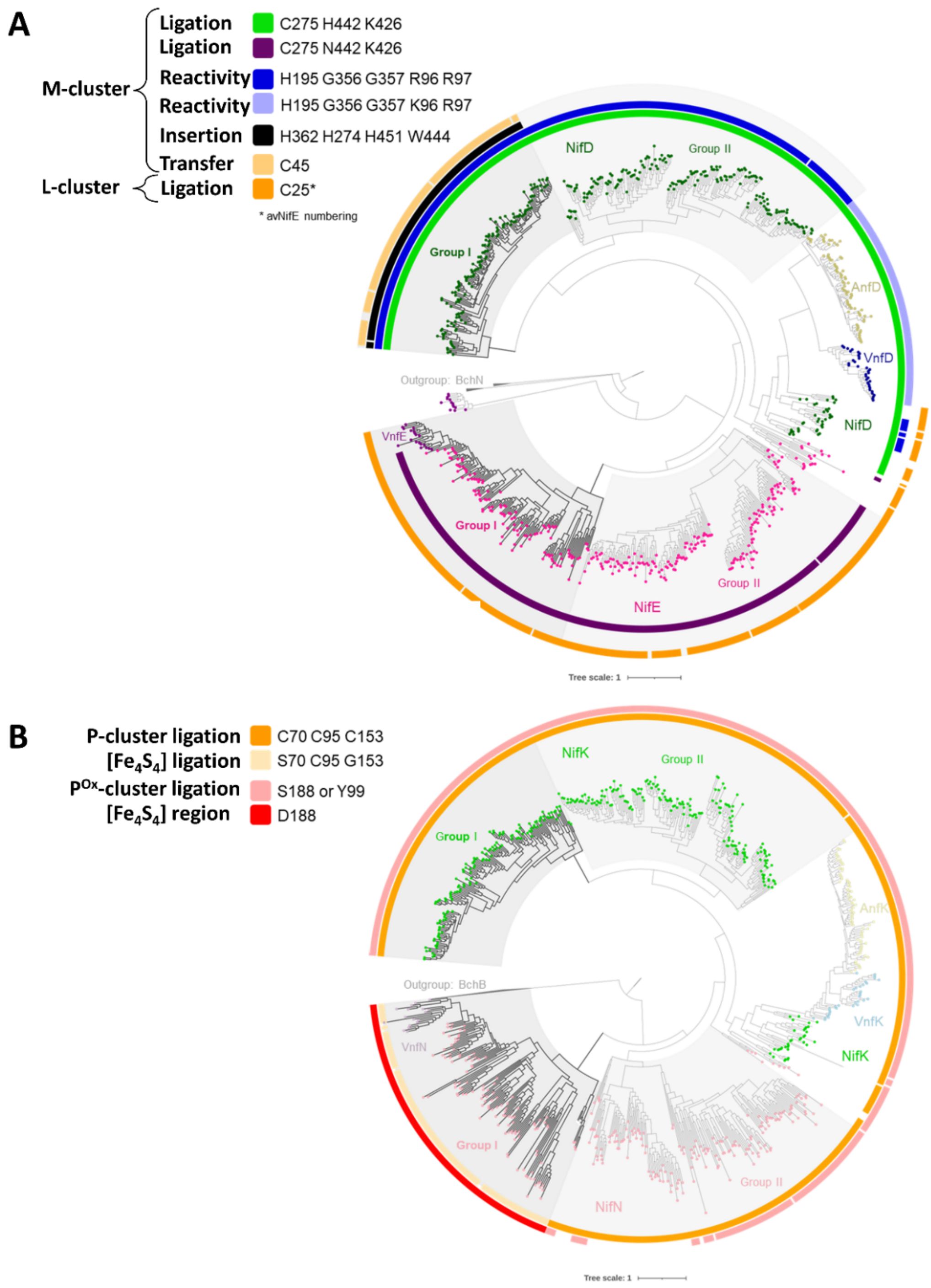

Fig. S4. Distribution of the M- and P-cluster ligands. The tree 1 reported in Ref. 14 was annotated in iTOL 6.8.1., rooted at midpoint, and pruned into α (**A**) and β (**B**) subtrees with outgroups collapsed for clarity. Sequence annotations are represented by colors of the nodes: (A) NifD, dark green; AnfD, beige; VnfD, navy blue; NifE, magenta; VnfE, violet; (B) NifK, green; AnfK, wheat; VnfK, light blue; NifN, light pink; VnfN, light purple. The presence of specific residues was extracted from the MSA file and marked with colors outside the trees as described in the figure. The clades belonging to group I and II are shown with shades of gray.

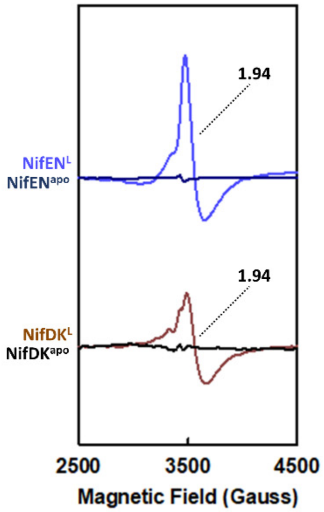

Fig. S5. EPR spectra of the IDS-oxidized NifEN and NifDK proteins used in this study. Shown are the spectra of NifEN^{L} (blue), NifEN^{apo} (dark blue), NifDK^{L} (brown), and NifDK^{apo} (black) proteins. The spectra of NifEN^{L} and NifDK^{L} are taken from Fig. 3B.

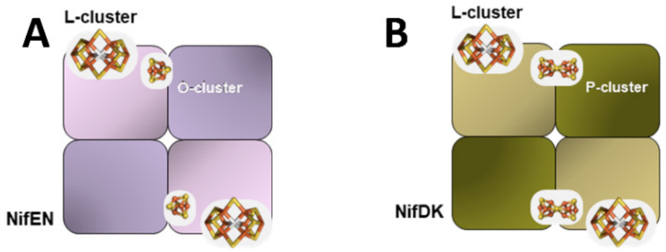

Fig. S6. Schematic presentations of NifDK^{L} and NifEN^{L}. (**A**) NifEN^{L} contains a surface exposed L-cluster that is ligated to the α-subunit of NifEN by Cys$^{\alpha 25}$ (*see* Fig. 1). (**B**) The kinetics of the L-cluster-specific Fe chelation of NifDK^{L} (*see* Fig. 3A) suggest that like NifEN^{L}, NifDK^{L} has its L-cluster ligated to the α-subunit by a conserved Cys$^{\alpha 45}$ at a surface-exposed location.

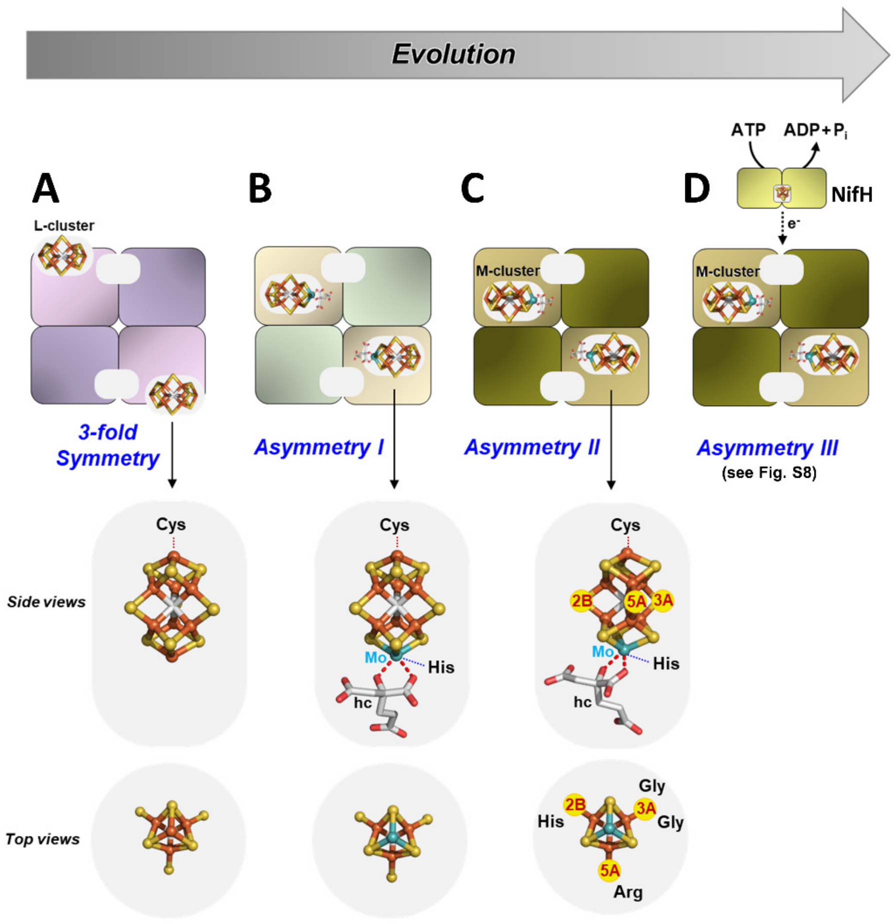

Fig. S7. Proposed evolution of the active-site cofactor of Mo-nitrogenase on a theme of asymmetry. (**A**) Based on our model, evolution of the active-site cofactor of nitrogenase begins with a surface-exposed, all-Fe cofactor (i.e., the [Fe_8S_9C] L-cluster) with a three-fold symmetry in terms of the three belt-sulfur sites. Such a symmetry can be best visualized in top view, along the Fe-C-Fe axis of the L-cluster. (**B**) Insertion of a heterometal (Mo) and an organic component (hc, homocitrate) at one end of the primitive, all-Fe cofactor does not change the overall geometry of the cofactor, yet it introduces a key asymmetric element (**Asymmetry I**) that not only modifies the redox characteristics of the cofactor, but also allows for the precise coordination of the two distinct ends of the cofactor (i.e., Mo/hc and Fe) by two specific ligands (i.e., histidine and cysteine) located at a more embedded cofactor binding site. (**C**) Breaking the three-fold symmetry of the cofactor further, the local protein environment evolves toward having different capacities for donating protons to the three belt-sulfur locations of the cofactor (**Asymmetry II**) that could potentially enable a stepwise reduction of N_2 at these positions, possibly facilitated by a rotation of the cofactor (*see* Fig. S8 for details) via an alternating elongation/breaking of the two Mo-O bonds between Mo and homocitrate. (**D**) Introduction of a specific reductase (NifH) as the obligate electron donor for the catalytic component (NifDK) renders the Mo-nitrogenase an efficient two-component system, with a plausible, alternating interaction of NifH with the two αβ-dimers of NifDK (**Asymmetry III**), resulting in an ATP-dependent, asynchronous rotation of the two cofactors of Mo-nitrogenase (*see* Fig. S8 for details).

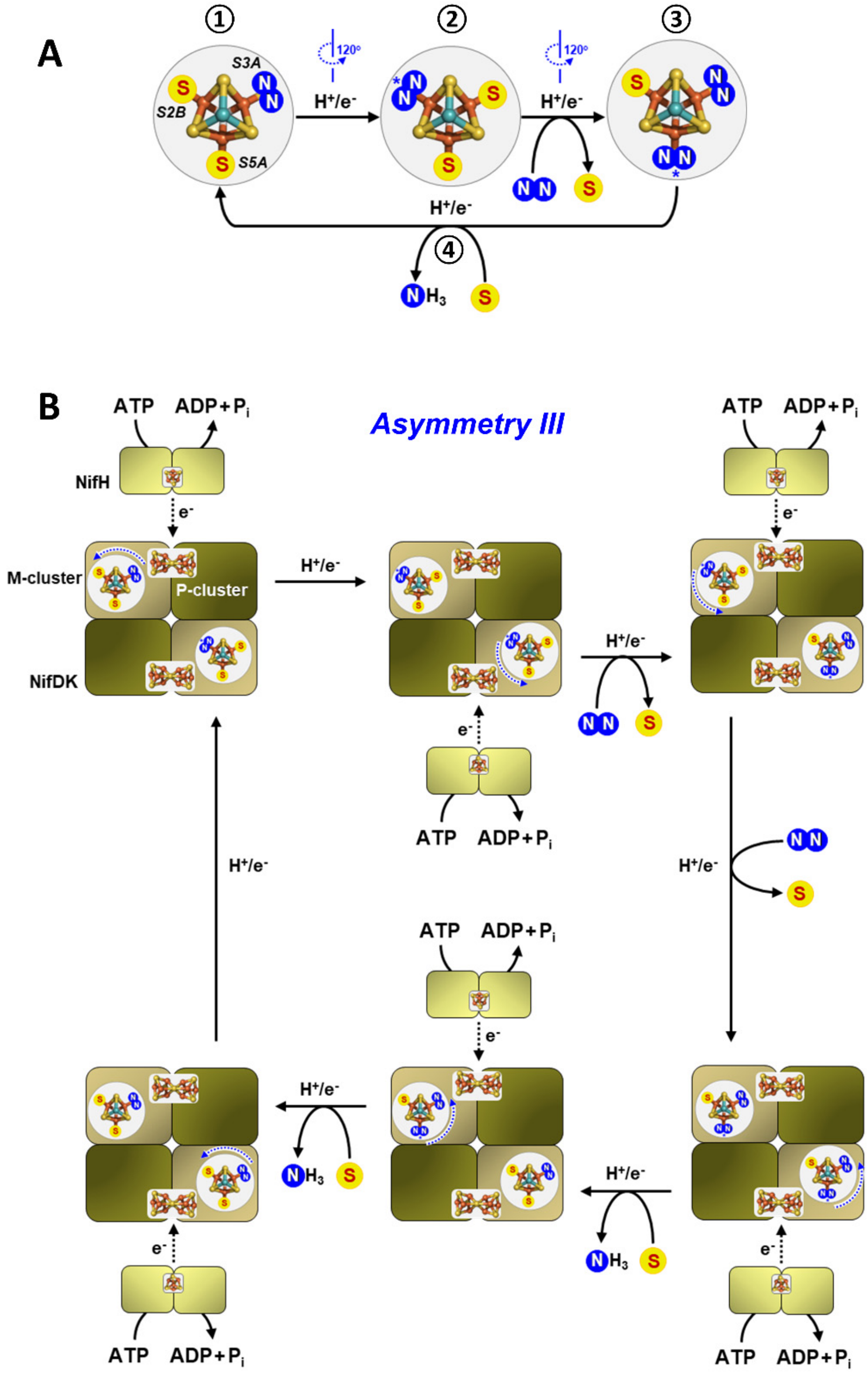

Fig. S8. Proposed model of stepwise N_2 reduction occurring in tandem via asynchronous cluster rotation. (**A**) Rotation of the M-cluster in the direction of S3A→S2B→S5A is presumably enabled by an alternating elongation/breaking (via protonation) of the two Mo-O bonds between Mo and homocitrate (*17, 18*). Such a rotation allows for binding of N_2 via belt-sulfur displacement at the S3A site (①) and stepwise reduction of N_2 to NH_3 at the S2B (②) and S5A (③) sites. Subsequently, a 'new' N_2 is bound at the S3A site via belt-sulfur displacement (③), followed by release of NH_3 from the S5A site via belt-sulfur replacement (④), and continuous cluster rotation that facilitates the next round of N_2 reduction. (**B**) An alternating docking of NifH on the two αβ-

dimers of NifDK (**Asymmetry III;** *also see* Fig. S7) results in an asynchronous rotation of the two M-clusters in NifDK and, consequently, permits the same sequence of events to occur one step apart at the two M-clusters. Such an alternating docking of NifH on NifDK drives ATP-dependent cofactor rotation via Mo-O bond elongation/breakage in one dimer while permitting the cofactor in the other dimer to 'idle' in one place long enough for the completion of a certain reaction step. This proposal is consistent with the cryo-EM observation of a 1:1 molar ratio between NifH and NifDK in a turnover complex (*25*) as well as the stepwise assembly of the two P-clusters of NifDK that involves an analogous asymmetric interaction between NifH and the two dimers of a P-cluster precursor containing form of NifDK (*20*).
